# Radiomics Nomogram of DCE-MRI for the Prediction of Axillary Lymph Node Metastasis in Breast Cancer

**DOI:** 10.3389/fonc.2020.541849

**Published:** 2020-10-27

**Authors:** Ning Mao, Yi Dai, Fan Lin, Heng Ma, Shaofeng Duan, Haizhu Xie, Wenlei Zhao, Nan Hong

**Affiliations:** ^1^ Department of Radiology, Yantai Yuhuangding Hospital, Qingdao University, Yantai, China; ^2^ Department of Radiology, Peking University Shenzhen Hospital, Shenzhen, China; ^3^ Precision Health Institution, GE Healthcare, China, Shanghai, China; ^4^ Department of Radiology, Peking University People’s Hospital, Beijing, China

**Keywords:** breast cancer, lymphatic metastasis, radiomics, nomogram, magnetic resonance imaging

## Abstract

**Purpose:**

This study aimed to establish and validate a radiomics nomogram based on dynamic contrast-enhanced (DCE)-MRI for predicting axillary lymph node (ALN) metastasis in breast cancer.

**Method:**

This retrospective study included 296 patients with breast cancer who underwent DCE-MRI examinations between July 2017 and June 2018. A total of 396 radiomics features were extracted from primary tumor. In addition, the least absolute shrinkage and selection operator (LASSO) algorithm was used to select the features. Radiomics signature and independent risk factors were incorporated to build a radiomics nomogram model. Calibration and receiver operator characteristic (ROC) curves were used to confirm the performance of the nomogram in the training and validation sets. The clinical usefulness of the nomogram was evaluated by decision curve analysis (DCA).

**Results:**

The radiomics signature consisted of three ALN-status-related features, and the nomogram model included the radiomics signature and the MR-reported lymph node (LN) status. The model showed good calibration and discrimination with areas under the ROC curve (AUC) of 0.92 [95% confidence interval (CI), 0.87–0.97] in the training set and 0.90 (95% CI, 0.85–0.95) in the validation set. In the MR-reported LN-negative (cN0) subgroup, the nomogram model also exhibited favorable discriminatory ability (AUC, 0.79; 95% CI, 0.70–0.87). DCA findings indicated that the nomogram model was clinically useful.

**Conclusions:**

The MRI-based radiomics nomogram model could be used to preoperatively predict the ALN metastasis of breast cancer.

## Introduction

Breast cancer is a malignant tumor that endangers women’s health and quality of life. Axillary lymph node (ALN) is the first station of breast lymphatic drainage, which collects approximately 75% of breast lymph. Thus, ALN is the most easily metastasized site of breast cancer. ALN status is an important factor affecting the treatment of patients with breast cancer and is assess by the gold standards ALN dissection and sentinel lymph node (LN) biopsy. However, ALN dissection is invasive and has many complications, such as lymphedema, and sentinel LN biopsy is also invasive (1). Therefore, a non-invasive prediction tool for preoperative LN status is needed.

MRI has been widely used in breast examination because of its good soft tissue contrast, high sensitivity, and high negative predictive rate (2). Although this technique is superior to digital mammography and ultrasonography, its efficacy in identifying malignant nodes is unsatisfactory (2–4).

Radiomics can extract massive image features; transform medical images into high-dimensional and exploitable data; and use artificial intelligence to combine medical images, genes, and huge clinical data to establish a model that supports clinical decision-making and quantify tumor heterogeneity (5–9). This method has good clinical prospects (9–11). The combined analysis of multiple features including clinical ones is the most promising approach, especially for the clinical management of tumors (12–16). Furthermore, nomograms, which allow the investigation of multiple features in parallel, transform complex regression equation into visual graphs (17–19).

This study aimed to develop and validate a radiomics nomogram model based on dynamic contrast-enhanced (DCE)-MRI and clinical risk factors to determine its potential in predicting ALN metastasis in patients with breast cancer.

## Materials and Methods

### Patients

This retrospective study was approved by the Institutional Review Board. Inclusion criteria were as follows: (a) patients with breast cancer confirmed by histopathological examination, (b) available clinical information, and (c) surgery conducted after MR scanning. Exclusion criteria were as follows: (a) patients who underwent preoperative neoadjuvant chemotherapy or radiotherapy, (b) patients who underwent biopsy prior to MR scanning, and (c) patients with other tumors and (d) non-mass lesions without delineate boundaries. The patients were divided into two independent sets, namely, training (200 patients) and validation sets (96 patients).

Clinical data were obtained through the medical record systems. All images were reviewed by two radiologists with at least 10 years of experience in imaging diagnosis, and the largest diameter of the tumors, apparent diffusion coefficient (ADC) value, enhanced features and the short diameter of the largest LN were recorded. MRI-reported LN status refers to the imaging-based diagnosed LN status according to the radiologist. T2WI and DCE-MRI series were used for ALN diagnosis. A patient’s LN status was classified as positive (cN+) if one or more ALNs found on MR images met any one of the following MRI features: 1) visible ALN >10 mm in a short diameter, 2) ratio of the longest to shortest axes < 1.6, 3) eccentric cortical thickening, and 4) loss of fatty hilum. Those who did not met the above mentioned criteria and met the above criteria but showed no difference in terms of number, size, or shape compared with the contralateral ALN, the LN status was assumed to be negative (cN0) (20–22). LN was classified as positive when at least one of the four criteria was satisfied. Agreement from MRI-reported LN-status analyzed from two observers was compared using κ statistic, in which 0 < κ ≤ 0.4 indicates poor agreement, 0.4 < κ < 0.75 indicates good agreement, and 0.75 ≤ κ < 1 indicates excellent agreement. All disagreements were resolved through consultation.

### Pathological Evaluation

Pathology is the gold standard for LN metastasis. Radionuclide and methylene blue were used as tracers to ensure that all sentinel LNs were removed. The patients were injected with radionuclide 2−3 h prior to surgery. After anesthetization, methylene blue was injected into the patient’s breast, which was then gently rubbed to allow the dye to further spread along the lymph vessels. Radionuclide detector was used to identify LNs labeled with nuclide during surgery. Stained LNs were also searched along the blue-stained lymph-vessels from top to bottom, inside to outside, and toward the axilla. All LN specimens were fixed by 4% neutral formaldehyde, embedded in paraffin, sectioned in 4-um thickness, sequentially sectioned, and stained by hematoxylin and eosin stain. Finally, the morphology of LN tissues was observed by two pathologists under BX53 electron microscope, and the tumor cells were confirmed as LN metastasis. If the pathological result of LN biopsy was inconsistent with that of surgery, then the latter was used as the standard.

Histopathological information, such as histological grade, estrogen receptor, progesterone receptor, human epidermal growth factor receptor type 2, and Ki-67, was obtained from the medical record system. Threshold values were ≤ 1% for the estrogen receptor and progesterone receptor levels and ≤ 20% for Ki-67 (23).

### MR Image Acquisition

**Figure 1:**
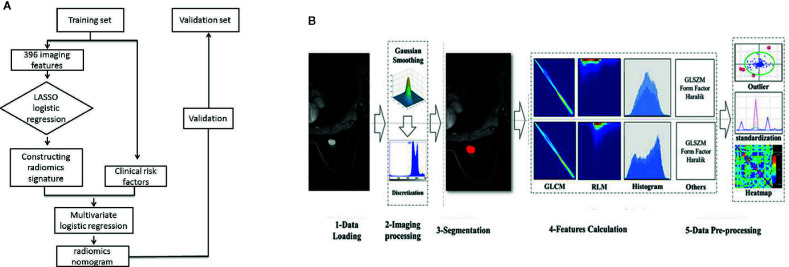
Study flowchart **(A)** and radiomics workflow **(B)**.

**Figure 1A** presents the study flowchart. All images were obtained on a 3.0T MRI system (GE Discovery 750W) using an eight-channel breast-dedicated coil in prone position. The MRI sequences included axial T1-weighted imaging, axial T2-weighted imaging, DCE-MRI, and sagittal contrast-enhanced imaging.

The scanning parameters were as follows: ① axial T1WI (TR = 460 ms, TE = 6.3 ms, slice thickness = 5 mm, slice spacing = 1 mm); ② axial fat suppression T2WI (TR = 5210 ms, TE = 84.7 ms, slice thickness = 5 mm, slice spacing = 1 mm); ③ axial DWI (SE-EPI sequence, TR = 2496 ms, TE = 71.9 ms, slice thickness = 5 mm, slice spacing = 1 mm, B = 0/800 s/mm2); ④ DCE scanning was performed on T1 fat suppression. The contrast medium was GD-DTPA, dose = 0.2 mmol/kg, TR = 5.7 ms, TE = 1.7 ms, slice thickness = 2 mm, slice spacing = 0 mm, FOV = 36 cm × 36cm, matrix = 288 × 320, phases = 8, and total time=6 min; ⑤ and sagittal contrast-enhanced imaging was performed after DCE (TR = 6.7 ms, TE = 1.7 ms, slice thickness = 2 mm, slice spacing = 0 mm, FOV = 28 cm × 28 cm, matrix = 200 × 256. Scan ranges for breast MRI were as follows: in the prone position, the bilateral breasts naturally hung over the center of the breast coil; in the horizontal axis position, the bilateral breasts were located in the center of the FOV, and the range included the entire bilateral breasts and bilateral axillary regions; and in the sagittal position, the positioning line was parallel to the long axis of the breast. All DICOM data were exported from Picture Archiving and Communication Systems.

### Image Preprocessing

Image preprocessing was necessary prior to feature extraction. This process consisted of three steps, namely, standardization of the gray value of the region of interest (ROI), discretization of the gray level, and image resampling (24–26).

### Image Segmentation and Radiomics Feature Extraction

**Figure 1B** presents the radiomics workflow. DCE-MRI (the peak enhanced phase of the multiphase contrast-enhanced MRI selected in accordance with time intensity curve) was selected for radiomics analysis, and the primary tumor was manually segmented using 3D-ROI by two trained radiologists with at least 10 years of experience in breast imaging and who were also blinded to the LN status and pathologic results. Twenty-four breast lesions were randomly selected to calculate the intra- and inter-observer agreement of the feature extraction. First, the two radiologists extracted the radiomics features. After 2 weeks, reader 1 used the same method to extract the radiomics features. Inter- and intra-correlation coefficients (ICCs) were calculated to assess the reproducibility of the radiomics features, and ICCs > 0.80 were considered as good agreement. The remaining image segmentation was performed by reader 1. Image segmentation and radiomics feature extraction were performed on Artificial Intelligence Kit software (version 3.2.0; GE Healthcare, Shanghai, China).

### Feature Selection and Radiomics Signature Building

The features with high repeatability (ICC > 0.80) were selected. Feature selection was then performed using the LASSO logistic regression method in the training set. This method is suitable for high-dimensional data (27). Radiomics score reflecting the risk of ALN metastasis was calculated for each patient by using a linear combination of selected features weighted by their respective coefficients. Receiver operator characteristic (ROC) curves were used to assess the performance of the radiomics signature in the two sets.

### Construction of Radiomics Nomogram

Clinical factors included age, tumor size, tumor margin, and MRI-reported LN status. Risk factors were determined by univariate and multivariate logistic regressions. Collinearity was assessed by variance inflation factor (VIF). Likelihood ratio test with backward step-down selection was applied for logistic regression. A nomogram was established in the training set on the basis of multivariable logistic regression.

### Assessment of Nomogram Performance

ROC curves were used to assess the predictive performance of the radiomics nomogram in the training set, and calibration curves were employed to evaluate the agreement between the observed and predicted results. Good agreement between the true state of ALN and the predicted probability based on radiomics nomogram was achieved when the calibration curves were close to the diagonal line. Hosmer–Lemeshow test was used to determine the goodness of fit of the radiomics nomogram.

### Validation of Radiomics Nomogram

The radiomics nomogram was validated using the validation set with the same formula in the training set. ROC and calibration curves were used to assess the predictive performance of the radiomics nomogram.

### Clinical Use

The clinical usefulness of the nomogram was assessed using decision curve analysis (DCA) in the validation set. The ROC curve was used to calculate the area under the ROC curve (AUC). However, ROC only considers the specificity and sensitivity of the method, and DCA determines the clinical practicability of radiomics nomograms by quantifying the net benefits under different threshold probabilities in the validation set. The calculation formula is as follows:

$$net\;benefit\;treated=\frac{TP}{n}-\frac{FP}{n}\left(\frac{P_{t}}{1-P_{t}}\right),$$

where *TP* and *FP* are the true positive count and the false positive count, respectively; and *n* is the number of subjects; and *P_t_* is the threshold probability.

### Statistical Analysis

All statistical tests were conducted in R3.5.1. Chi-square or Fisher’s exact test was used to compare the differences in categorical variables, and a two-sample t test was applied to compare the differences in age and tumor size. LASSO logistic regression was used to select the most discriminating features and build the radiomics signature *via* 10-fold cross validation based on the minimum criteria. The radiomics signature was calculated by combining the features weighted by their coefficients. Clinical factors were used to construct the clinical model by using multivariable stepwise-backward logistic regression, and the clinical nomogram was provided. VIFs were accessed to exclude multi-collinearity, and the combined nomogram was built similarly to clinics, except for the combination of clinical factors and radiomics signature. ROC analysis, calibration curve, and DCA analysis were employed to evaluate the performance of the nomograms. DeLong’s test was used to compare the differences of ROC curves. In addition, “glmnet,” “glm,” “rms,” “pROC,” “Calibration Curves,” and “Decision Curve” packages were used. P < 0.05 indicates statistically significant difference.

## Results

### Patient Characteristics

**Table 1** exhibits the patients’ characteristics in the training and validation sets. The kappa value obtained in agreement of observation is 0.85, indicating a good agreement between two observers in MRI-reported LN status classification. Molecular subtype was detected in both groups, which showed no significant differences between the metastatic and non-metastatic groups in terms of age, tumor size, ADC value, enhanced features and histological grade (p > 0.05). The proportions of ALN metastasis in the training and validation sets were 47.1% and 47.3%, respectively. These results justified their use as training and validation sets.

**Table 1:** Patient characteristics in the training and validation sets.

	Training set (N = 200)		p value	Validation set (N = 96)		p value
	pN+	pN0		pN+	pN0	
**Age**, **years (SD)**	49.10 ± 10.1	48.10 ± 11.0	0.642	49.50 ± 9.10	49.51 ± 11.5	0.752
**Tumor size, cm (SD)**	2.58 ± 0.9	2.48 ± 1.3	0.576	2.51 ± 1.3	2.51 ± 1.2	0.677
**ADC value (SD)**	0.87 ± 0.2	0.84 ± 0.2	0.196	0.88 ± 0.2	0.85 ± 0.2	0.296
**Enhancement**						
Even	7	6	0.101	4	3	0.123
Uneven	67	69		37	28	
Ringlike	26	25		13	11	
**TIC**			0.501			0.396
Type I	6	5		4	3	
Type II	41	39		20	18	
Type III	53	57		30	21	
**Histological grade**						
I	10	8	0.152	6	4	0.153
II	40	46		20	22	
III	50	46		28	16	
**Molecular subtype**			0.156			0.149
Luminal A	70	76		42	34	
Luminal B	8	10		6	2	
HER2 over-expression	16	10		5	4	
Basal like	6	4		1	2	
**Ki-67 status**			0.316			0.322
Positive	82	79		44	34	
Negative	18	21		10	8	

pN+, pathologically confirmed lymph node positive; pN0, pathologically confirmed lymph node negative; ADC, apparent diffusion coefficient; TIC, time signal intensity curve.

### Feature Selection, Radiomics Signature Building, and Validation

**Figure 2:**
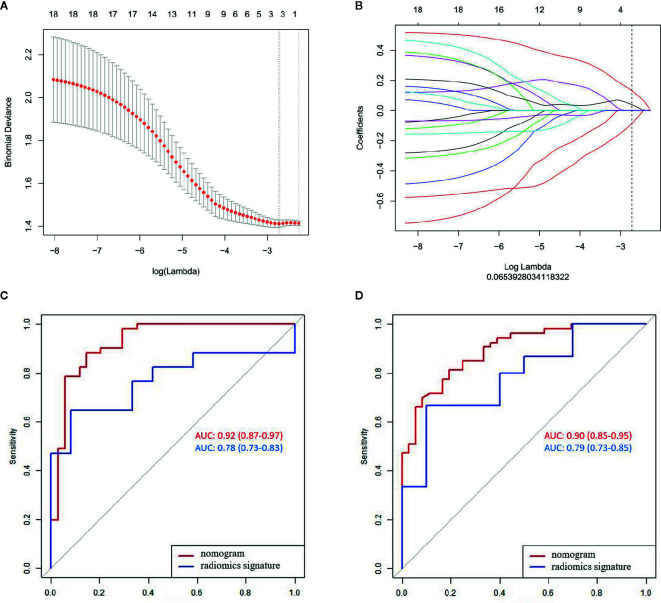
LASSO algorithm for radiomics feature selection and the predictive performance of the radiomics. **(A)** Mean square error path using 10-fold cross validation; **(B)** LASSO coefficient profiles of the radiomics features; and **(C, D)** ROC curves of the radiomics signature and nomogram in the training and validation sets.

A total of 396 radiomics features were extracted from each MR image and divided into six groups, namely, histogram, form factor matrix, gray-level co-occurrence matrix (GLCM), gray-level size zone matrix (GLSZM), Haralick matrix, and run length matrix (RLM). The ICCs ranged from 0.863 to 0.982 and from 0.832 to 0.935 in the intra- and inter-observers, respectively. Three LN state-related features with non-zero coefficients, namely, GLCMEnergy_AllDirection_offset7, LargeAreaEmphasis, and Correlation_AllDirection_offset7_SD were selected from the LASSO model in the training set (**Figures 2A, B**). The calculation formula is as follows:

$$\begin{array}{c} Rad-score\;=\;-0.292\\ +\left(-0.501\times Correlation\_AllDirection\_offset7\_SD\right)\\ +\;\left(-0.0551\times \;GLCMEnergy\_AllDirection\_offset7\right)\\ +\left(-0.0821\times \;LargeAreaEmphasis\right) \end{array}$$

Significant difference was observed in the radiomics scores between LN-negative and LN-positive patients in the two sets (P < 0.01). The radiomics signature yielded AUCs of 0.78 (95% CI, 0.73–0.83) in the training set and 0.79 (95% CI, 0.73–0.85) in the validation set (**Figures 2C, D**).

### Development and Validation of the Radiomics Nomogram Model

**Figure 3:**
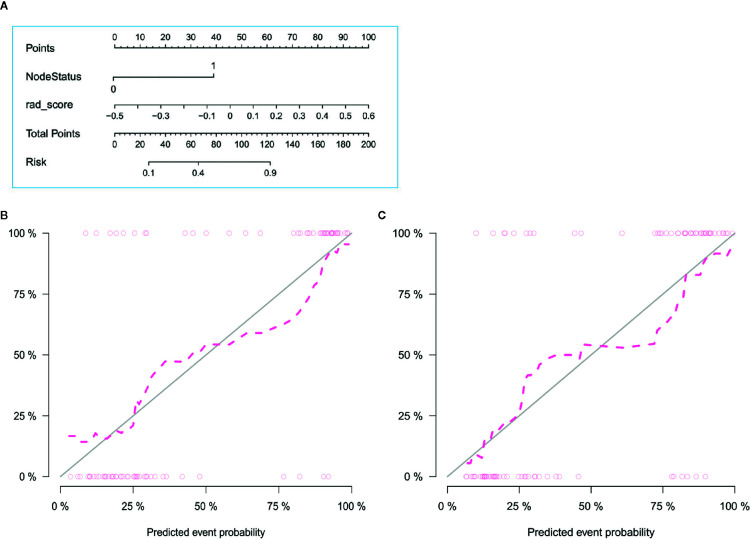
Radiomics nomogram with radiomics signature and LN status **(A)** and calibration curves of the radiomics nomogram in the training **(B)** and validation **(C)** sets. Calibration curves indicate that the predicted probability has a good agreement with the actual state of axillary lymph node.

**Figure 4:**
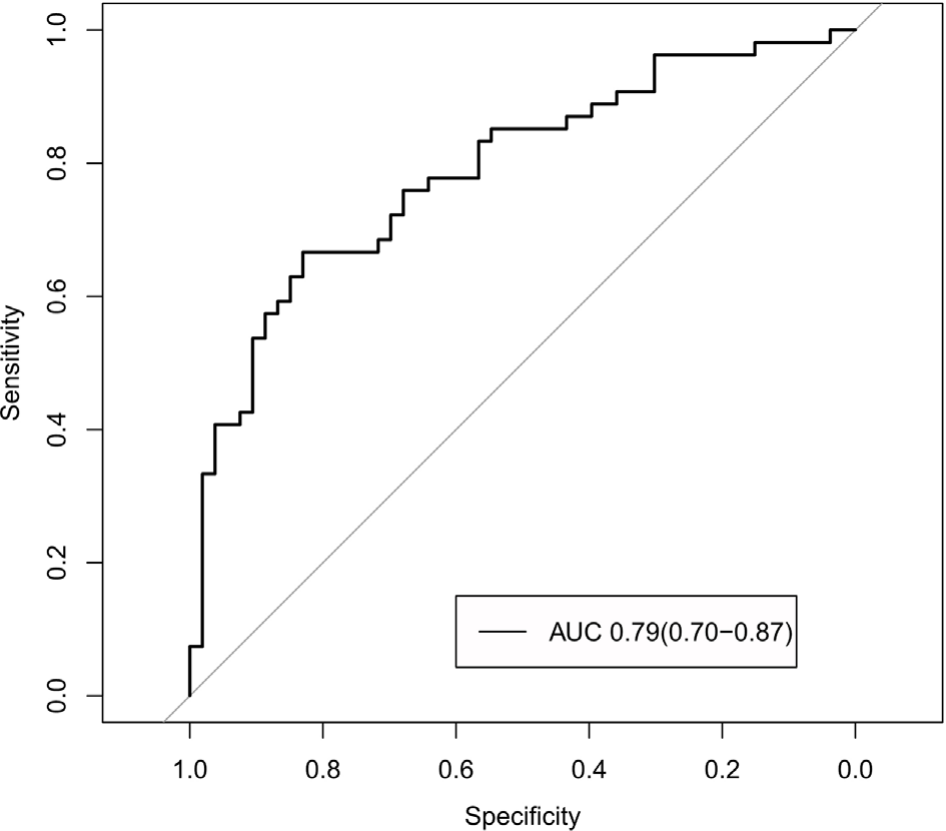
ROC curves of the nomogram in the cN0 subgroup in the validation set.

**Figure 5:**
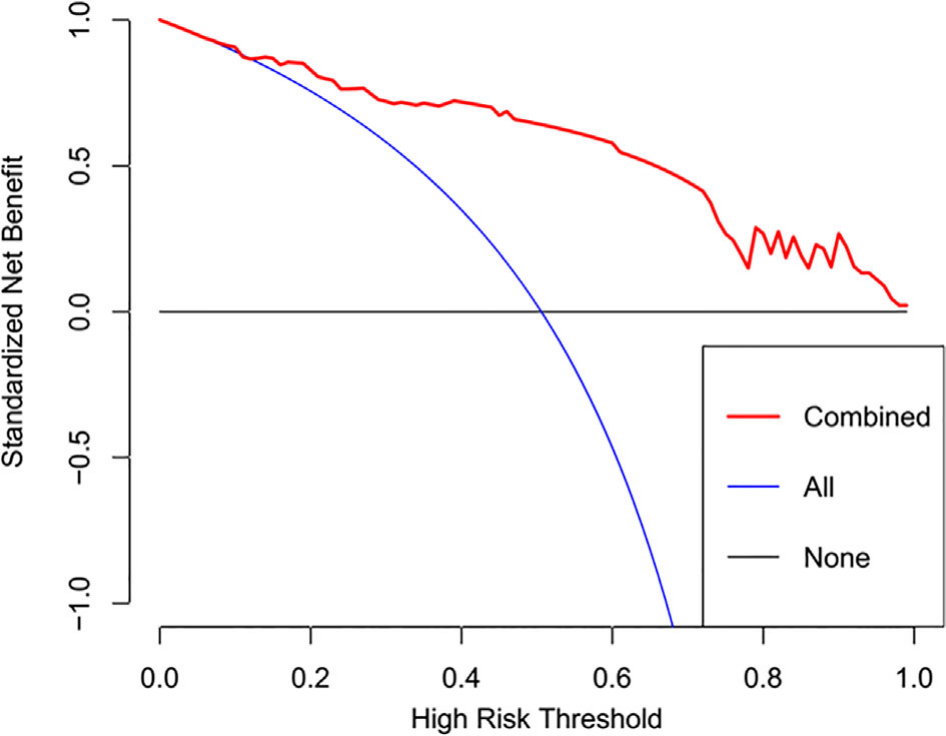
DCA of the radiomics nomogram. The y axis represents the net benefits, while the x axis represents the threshold probability. The red line represents the radiomics nomogram. The blue line represents the assumption that all patients were included in the lymph node metastasis group. The black line represents the assumption that all patients were included in the non-lymph node metastasis group.

The radiomics signature and the MRI-reported LN status were identified as risk factors of LN metastasis in breast cancer (**Table 2**). The MRI-reported LN status was a qualitative feature that could be easily obtained. No collinearity was observed because the VIF of the predictor ranged from 1.10 to 1.25. The nomogram model included the radiomics signature and the MRI-reported LN status (**Figure 3A**). In the calibration curve in **Figures 3B, C**, the gray line represents perfect prediction, and the dotted line represents the calibration curve of the radiomics nomogram. The calibration curve and the nonsignificant Hosmer–Lemeshow test showed good agreement between the true state of ALN and the predicted probability based on radiomics (P = 0.663). The radiomics nomogram yielded AUCs of 0.92 (95% CI, 0.87–0.97) in the training set and 0.90 (95% CI, 0.85–0.95) in the validation set (**Figures 2C, D**). Significant difference was observed between the differences of ROC curves in the two sets (P < 0.001). The nomogram model yielded an AUC of 0.79 (95% CI, 0.70–0.87) in the cN0 subgroup (**Figure 4**). The results of DCA are shown in **Figure 5**. When the threshold probability ranged from 0.1 to 1.0 in the validation set, the radiomics nomogram to predict LN metastasis provides more net benefit than the “treat all” or “treat none” scheme. Therefore, our nomogram excellently performed in discrimination, calibration, and clinical use.

**Table 2:** Risk factors for ALN metastasis in breast cancer.

Variable	Univariate logistic regression		Multivariate logistic regression	
	OR (95% CI)	*p*	OR (95% CI)	*p*
Radiomics score	2.711 (1.778–4.480)	<0.001*	2.757 (1.856–4.389)	<0.001*
Age, years	0.987 (0.953–1.021)	0.545	NA	NA
Tumor size, cm	1.113 (0.688–1.424)	0.927	NA	NA
Tumor margin	0.848 (0.357–1.788)	0.699	NA	NA
LN status	2.286 (1.262–4.265)	0.015*	2.110 (1.135–3.897)	0.016*

OR, odds ratio; NA, not available. These variables were eliminated in the multivariate logistic regression model in the training set; thus, the OR and p values were not available. *p < 0.05.

## Discussion

LN metastasis is a negative prognostic factor of breast cancer (28, 29). Thus, non-invasive LN assessment tools are promising. In this study, a radiomics nomogram model based on MRI was developed to predict the pretreatment of ALN metastasis in breast cancer and was validated using an independent dataset. This nomogram model was composed of radiomics signature and MR-reported LN status with AUCs of 0.92 in the training set and 0.90 in the validation set. LN metastasis has been predicted on the basis of clinical information or radiomics features only (2, 17, 30, 31). This research combined clinical information with radiomics features and used visualization nomogram to predict LN metastasis.

LN status has a certain diagnostic performance in differentiating ALN metastases (22). In this study, the MRI-reported LN status remarkably differs between the metastatic and non-metastatic groups. Moreover, univariate and multivariate logistic regression models have identified the MRI-reported LN status as an independent predictor of ALN metastasis. Therefore, this status was used as a predictor of the model. Previous study (32) used dynamic gadopentetate dimeglumine (Gd) enhanced MRI to evaluate axilla status in patients with breast cancer, and used ROC curves to compare enhancement indices and nodal area with histopathology of excised nodes, with AUCs from 0.77 to 0.88. Our results showed that compared with conventional MRI, radiomics nomogram had higher AUC of 0.90.

The application of radiomics nomogram provides a new approach for establishing a LN metastasis prediction model by using multiple characteristics. We previously used CESM-based radiomics signature and CESM-reported LN status to construct a radiomics nomogram to predict axillary LN metastasis, yielding an AUC of 0.79 in external validation cohort (33). Qiu et al. used 21 texture features derived from ultrasound imaging and ultrasound-reported LN status to predict LN metastasis in breast cancers, with an AUC of 0.759 in validation set (34). In our present study, the proposed MRI-based radiomics nomogram showed better performance than CESM-based and ultrasound-based radiomics nomogram, which may be used as an individualized model to visualize the risk of ALN metastasis by doctors and patients, and may meet the requirements for the development of precision medicine (35).

Tan et al. (36) not only used radiomics signature to predict LN status but also incorporated molecular subtype and PR status in nomogram. Other previous studies also used clinic-pathological characteristics to establish models in predicting LN metastasis of breast cancer patients, such as lymphovascular invasion and serum miRNA expression (37, 38), which might have a limited clinical implication, because characteristics such as molecular subtype, PR status, lymphvascular invasion, and miRNA was usually obtained by biopsy or other examinations, which to some extent limited the clinical application of these prediction models. However, the proposed radiomics nomogram only incorporated the MR-reported LN status and radiomics signature, which could be obtained by a non-invasive way before surgery, with an acceptable performance in LN metastasis predicting.

The discrimination and calibration performance of radiomics nomograms does not represent their clinical usefulness. Thus, whether this technique could improve patient outcome was assessed using DCA. Within the threshold probability range of 0.1–1.0, the radiomics nomogram provided more net benefits than the “treat all” or “treat none” scheme.

The proposed nomogram model showed good discriminating performance in cN0 patients who are difficult to diagnose by using traditional methods.

This study offered other notable advantages. Prior to feature extraction, some preprocessing techniques were applied to improve feature discrimination, and ICCs were used to evaluate the reproducibility of the radiomics feature extraction. These methods improved the reliability of this study.

This study has several limitations. First, the patients were enrolled from a single institution with a limited number. Despite the promising prospect, a large sample size and a multicenter study are warranted to prove the robustness of the proposed nomogram. Second, image segmentation was conducted manually. Although ICCs exhibited good reproducibility in feature extraction, the automated method for image segmentation provides stability (39, 40). Third, the methodology was limited by its statistical robustness, which could be overcome only through the true-blinded testing of the hypothesis. Future studies should adopt a double-blinded prospective design. Fourth, this study was performed retrospectively. In the future, the authors aim to collaborate with surgical colleagues and develop a prospective study to validate the proposed nomogram. Finally, the radiomics features were not extracted from the LNs.

In summary, the radiomics nomogram combined with MRI-based radiomics and clinical risk factors exhibited good predictive performance, calibration, and clinical utility in identifying ALN metastasis in patients with breast cancer. MRI-based radiomics could serve as a potential tool to help clinicians generate optimal clinical decisions and avoid overtreatment for patients with breast cancer.

## Data Availability Statement

All datasets generated for this study are included in the article/supplementary material.

## Ethics Statement

The studies involving human participants were reviewed and approved by the Institutional Review Board of Yantai Yuhuangding Hospital, Affiliated Hospital of Qingdao University. Written informed consent for participation was not required for this study in accordance with the national legislation and the institutional requirements.

## Author Contributions

NM and YD created the study design. HX, HM, and FL collected the data. NM and SD processed the data. NH and WZ conducted data analysis. NM and YD wrote the manuscript. All authors contributed to the article and approved the submitted version.

## Funding

This study was supported by the National Natural Science Foundation of China (82001775).

## Conflict of Interest

SD was employed by the company GE Healthcare.

The remaining authors declare that the research was conducted in the absence of any commercial or financial relationships that could be construed as a potential conflict of interest.
